# Differential Requirement of Vav Proteins for Btk-dependent and –Independent Signaling During B Cell Development

**DOI:** 10.3389/fcell.2022.654181

**Published:** 2022-02-23

**Authors:** Annika C. Betzler, Sebastian Kieser, Katja Fiedler, Simon Laban, Marie-Nicole Theodoraki, Patrick J. Schuler, Thomas Wirth, Kerry Tedford, Klaus-Dieter Fischer, Thomas K. Hoffmann, Cornelia Brunner

**Affiliations:** ^1^ Department of Oto-Rhino-Laryngology, Head and Neck Surgery, Ulm University Medical Center, Ulm, Germany; ^2^ Institute for Physiological Chemistry, Ulm University, Ulm, Germany; ^3^ Institute of Biochemistry and Cell Biology, Medical Faculty, Otto-von-Guericke University, Magdeburg, Germany

**Keywords:** BTK, bruton’s tyrosine kinase, VAV, B cell development, Ca2+ siganling, secondary lymphoid organs

## Abstract

Btk and Vav proteins are all components of the signalosome that builds upon B cell receptor (BCR) activation. However, the role of Vav proteins within the signalosome is quite complex and not yet fully understood. Until now, studies of these have focused predominantly on a deficiency of Vav proteins alone or in combination with other Vav protein family members. Since a physical association of Btk with Vav was shown previously, we asked whether these molecules lie in the same or independent signaling pathways. By analyzing Vav1 and Vav3 single knock-out mice and generating double-knock-out animals deficient for either Vav1 or Vav3 and Btk, we observed, in line with previous publications, no severe B cell developmental defects when either Vav1 or Vav3 alone are not expressed. However, a simultaneous deficiency of Btk together with either Vav1 or Vav3 leads to a severe reduction of splenic B cells, which exhibit an immature phenotype. B cell developmental defects of Btk/Vav1-double deficient mice in the periphery were more severe than those observed in Btk-single-deficient animals. Additionally, morphological changes in splenic microarchitecture were observed in double- but also in single-knock-out mutants. These observations were accompanied by reduced BCR-induced Ca^2+^ mobilization, proliferation, germinal center formation and immunoglobulin secretion. Although deletion of Btk alone impaired Ca^2+^ mobilization upon BCR activation, the defect was even more severe when Vav1 or Vav3 were also mutated, indicating that Btk and the Vav proteins act in separate pathways that converge on Ca2+ signaling. *In vitro* ASC differentiation suggests that both B and T cells contribute to the observed phenotype of a Btk/Vav-double deficiency. Our results show that Vav proteins and Btk are both components of the BCR-activated signalosome but control separate signaling pathways important for B cell development.

## Introduction

The non-receptor tyrosine kinase Btk (Bruton’s Tyrosine Kinase) plays a central role for immune cells of the innate as well as the adaptive immune system (Brunner et al., 2005; Weber et al., 2017). However, one of the most striking consequences of a defective Btk function caused by mutations along the Btk genomic locus in humans concerns the B cell compartment leading to X-linked Agammaglobulinemia (XLA). XLA is characterized by the complete failure to generate a functional peripheral B cell pool. Affected patients show arrested B cells at the pre-B-cell stage in the bone marrow and consequently an almost complete loss of normal B cell numbers in the peripheral circulation, leading to the absence of primary and secondary immunoglobulins (Conley, 1985; Campana et al., 1990; Winkelstein et al., 2006). A corresponding mouse model (Kerner et al., 1995; Khan et al., 1995) allows the detailed investigation of Btk function for the immune system, although the B cell phenotype is somewhat milder in mice. The serious consequences caused by a non-functional Btk are based on its central role in B-cell receptor (BCR) mediated signal transduction (Aoki et al., 1994; De Weers et al., 1994; Saouaf et al., 1994), as it is essential at multiple checkpoints of B cell differentiation and function (Turner et al., 2020).

Upon antigen-dependent B cell activation, the Src family kinase Lyn phosphorylates the immunoreceptor tyrosine-based activation motifs (ITAMs) of the BCR-associated dimeric molecules Igα and Igβ (CD79a and CD79b), an essential prerequisite for the recruitment of Syk to these sites (Rolli et al., 2002). Lyn also phosphorylates the cytoplasmic tail of the BCR co-receptor CD19 (Roifmann and Ke, 1993; van Noesel et al., 1993), necessary for the docking and activation of PI3K (Lien et al., 2017). PI3K activity requires phosphorylation by Syk (Beitz et al., 1999), which enables the PI3 mediated generation of PIP3 from PI-4,5-P_2_. This in turn allows the binding of Btk via its PH-domain to the plasma membrane and its subsequent activating phosphorylation by Lyn and Syk (Seda and Mraz, 2015).

Phosphorylated Igα serves also as a docking site for the adaptor protein SLP-65/BLNK (Src homology 2 domain-containing leukocyte protein of 65 kDa/B cells linker protein) (Engels et al., 2001). SLP-65/BLNK further facilitates the recruitment of a signalosome complex, formed by PLCγ2 (phospholipase Cγ2), Btk (bruton’s tyrosine kinase), Vav-proteins and PI3K (phosphoinositol 3 Kinase), regulating PLCγ2 activity in B cells (Chiu et al., 2002; Hendriks et al., 2014). The main role of Btk is to phosphorylate and thereby activate PLCγ2 (Takata and Kurosaki, 1996; Tomlinson et al., 2001; Watanabe et al., 2001; Kim et al., 2004). The lipase activity of PLCγ2 cleaves PIP2, leading to the generation of second messengers, inositol triphosphate (IP3) and diacylglycerol (DAG). These second messengers further activate different but linked signaling pathways implicated in the regulation of intracellular Ca^2+^ levels as well as in the activation of the MAPK (mitogen activated protein kinases) pathway (Hendriks et al., 2014; Seda and Mraz, 2015).

The importance of Vav proteins for B cell receptor signaling became obvious in studies of Vav-mutant mice, revealing a phenotype resembling that of BLNK, PI3K-p85 subunit, Btk, or PLCγ2 deficiency (Tarakhovsky et al., 1995; Zhang et al., 1995; DeFranco, 2001; Tedford et al., 2001). Vav proteins (Vav1, Vav2 and Vav3) belong to the family of guanosine nucleotide exchange factors (GEFs) and are characterized by the presence of a Dbl-homology (DH) domain (Rodríguez-Fdez and Bustelo, 2019). Upon signaling activation, these enzymes catalyze the guanosine diphosphate (GDP) to guanosine triphosphate (GTP) exchange in Rho proteins, inducing their rapid conformational changes from an inactive into the active GTP-bound state. Although all three Vav protein family members are inducible phosphorylated B cells, their contribution to the BCR-dependent signaling is different (Rodríguez-Fdez and Bustelo, 2019). However, the role of Vav proteins within the signalosome is quite complex and not yet fully understood. Beside their role in Ras-MAPK signal transduction, they are involved in the regulation of Ca^2+−^mediated signaling (Inabe et al., 2002; Vigorito et al., 2004).

Vav1 and Vav3 were shown to be implicated in Ca^2+^ mobilization upon BCR activation, whereas the Vav2 contribution to Ca^2+^ was rather modest (Tedford et al., 2001) or not seen (Löber et al., 2020). Additionally, in Vav2-deficient mice, B cell development and maturation is normal. Since the loss of a functional Btk leads to impaired intracellular Ca^2+^ mobilization accompanied by severe B cell developmental and functional defects, our study focused on the potential functional interaction of Btk with Vav1 and Vav3. Btk interacts directly with Vav (Guinamard et al., 1997) and both are part of the BCR-induced signalosome (Seda and Mraz, 2015), therefore we asked whether these molecules act in the same or in independent signaling pathways. Our comparative analyses of B cell development and function in either Btk-, Vav1- Vav3-single deficient and Btk/Vav1-or Btk/Vav3-double deficient mice revealed that Btk, Vav1 and Vav3 are involved in different signaling pathways important for B cell development and function.

## Materials and Methods

### Mice

Knockout mice for Btk (Khan et al., 1995), Vav1 (Fischer et al., 1998) and Vav3 (Luft et al., 2015) have been described previously. Single and double-deficient mice and wild type littermates (all at the C57BL/6J background) were used at age of 7–16 weeks. Btk-single and Btk/Vav3-double deficient mice were analyzed up to the age of 29 weeks. All animal experiments were in accordance with institutional guidelines and German animal protection laws (reference number: 35/9185.81-3/1555). Mice were maintained in a specific pathogen-free facility under standard housing conditions with continuous access to food and water. Mice used in the study were maintained on 12 h light, 12 h dark light cycle (6:00–18:00).

### Flow Cytometric and Calcium Flux Analyses

Blood as well as single cell suspensions of spleen and bone marrow (BM) were used for flow cytometric analysis. For surface staining, 25 µl blood diluted with 75 µl PBS/1 mM EDTA or cell pellets of spleen and BM resuspended in 100 µl of PBS with 0.5% BSA were stained with respective fluorophore-conjugated antibodies and incubated for 30 min at 4°C in the dark. Blood cells were then washed with PBS/1 mM EDTA and spleen and BM cells with PBS with 0.5% BSA, centrifuged at 1200 rpm at 4°C and resuspended in 300 µl PBS/1 mM EDTA or PBS with 0.5% BSA for flow cytometric analysis. Data were acquired on a Gallios cytometer (Beckman Coulter, United States) and analyzed with Kaluza Analysis software, version 2.1 (Beckman Coulter, United States). Calcium influx into B220^+^ B cells was analyzed as described previously (Brunner et al., 2003) and analyzed with a FACsort™ plus cytometer (Becton Dickinson; United States) and FlowJo analysis software.

### Hematoxylin-Eosin Staining

Spleens of mice were collected and put in 2.5–3.7% paraformaldehyde solution for 24 h and subsequently embedded in paraffin. 3.5 µm thick tissue sections were stained with Mayer’s hemalaun (4 min) and eosin (3 min). Specimens were then dehydrated and cover-slipped. Number and size of follicles was determined within a section of 2,41 mm^2^ using Zeiss Observer.D1 and the AxioVision (Carl Zeiss, Germany) software. Three image sections per animal were analyzed.

### Immunohistochemistry

Paraffin-embedded sections of spleens from immunized mice were stained with biotinylated Peanut Agglutinin (PNA) (Vector Laboratories, United States). This was followed by detection with horseradish-peroxidase conjugated streptavidin and AEC chromogen. Subsequently, slides were counter stained with hematoxylin. Number of follicles and Germinal Centers of the whole section were counted and the ratio of total Germinal Center number/total follicle number was determined. Three immunohistochemical images of spleen samples of each animal were used to measure GC size. Every PNA^+^ Germinal Center was identified manually and measured in ImageJ to calculate their size.

### Proliferation Assay

A single cell suspension of mouse splenocytes was prepared. Splenocytes were stained with 5 µM CFSE using CellTrace™ CFSE Cell Proliferation Kit according to the manufacturer’s protocol (Life Technologies, United States). CFSE-labeled cells were stimulated with 5 μg/ml anti-µ F (ab)2 (Jackson ImmunoResearch Laboratories, United States) to stimulate B cell proliferation and cultured for 7 days. Percent of B cell proliferation was determined by CFSE dilution using flow cytometry gating on B220^+^ B cells. Data were acquired on a Gallios cytometer (Beckman Coulter, United States) and analyzed with Kaluza Analysis software, version 2.1 (Beckman Coulter, United States).

### Western Blot

B cells were isolated using B cell isolation kit (Milteny Biotec, Germany) and stimulated with 20 μg/ml anti-µ F (ab)2 (Jackson ImmunoResearch Laboratories, United States) for 3 min. The following antibodies were used: anti-phospho-Akt (S473), anti-Akt, anti-phospho-PLCγ2 (Y1217), anti-PLCγ2 (all Cell Signalling, United States), anti-phospho-ERK (Y204), anti-ERK and anti-phospho tyrosine (all Santa Cruz Biotechnology, United States). Bands of interest were quantified, and in-lane background was subtracted. To determine specific phosphorylation level, the signal from phosphorylated band was divided by the signal from the appropriate loading control, and all values were normalized to the level of unstimulated wild type.

### Immunization

To test T-dependent antibody responses and Germinal Center reaction, mice were immunized with 10^8^ Sheep Red Blood Cells (SRBCs) (Cedarlane Laboratories, Canada) in PBS by i.p. injection. Mice were boosted after 14 days and analyzed 21 days after the first immunization. To asses antigen-specific antibody levels, mice were immunized with 100 µg NP-KLH (Biosearch Technologies) emulsified in Imject Alum (Thermo Fisher Scientific) by i.p injection. Serum was analyzed for the presence of antigen-specific immunoglobulins after 28 days.

### Enzyme-Linked Immunosorbent Assay

Total IgM and IgG antibody levels were detected in serum of mice by ELISA 21 days after immunization. 96-well plates were coated with 2 μg/ml of goat anti-mouse IgM, IgG1, IgG2b or IgG3 (SouthernBiotech, United States) overnight at 4°C. Subsequently, plates were blocked with 10% FCS in PBS overnight at 4°C. Sera samples were added at three different dilutions. Bound antibodies were detected using alkaline phosphatase-conjugated goat anti-mouse IgM, IgG1, IgG2b or IgG3 (SouthernBiotech, United States) diluted 1:1,000. 4-nitrophenil phosphate-disodium salt (Serva, Germany) was used as substrate. IgM and IgG serum titers were determined by using respective isotype-specific Ig standards (SouthernBiotech, United States). For determination of NP-specific antibody levels in sera from 28 d NP-KLH immunized mice. ELISA plates were coated overnight at 4°C with 10 μg/ml NP(9)- and NP(27)-BSA. The next day, plates were blocked with 5% BSA in PBS overnight at 4°C. Subsequently, plates were washed and serum samples were serially diluted in duplicate and incubated overnight at 4°C. Plates were then washed and incubated with alkaline phosphatase-conjugated goat anti-mouse IgM, IgG1 or IgG3 (SouthernBiotech, United States) diluted 1:1,000 for 1 h at 37°C. 4-nitrophenil phosphate-disodium salt (Serva, Germany) was used as substrate. Values were reported as relative absorbance.

### ASC Differentiation

B cells were isolated using B cell isolation kit (Milteny Biotec, Germany) and cultured in RPMI 1640 medium (Gibco) with 10% FBS, 1% penicillin/streptomycin and 50 µM β-mercaptoethanol. 3 × 10^6^ cells were cultured in 3 ml medium supplemented with 10 μg/ml anti-CD40 antibody (Milteny Biotec, Germany) and 30 ng/ml IL-4 (PeproTech, Germany) for 7 days. On day 4, cells were collected and analyzed for the presence of CD138^+^ B220^low^ ASCs by flow cytometry. On day 7, supernatants were harvested. To determine immunoglobulin levlels, 96-well plates were coated with 2 μg/ml of goat anti-mouse IgM and IgG1, overnight at 4°C. Subsequently, plates were blocked with 10% FCS in PBS for 2 h at RT. Supernatants were added in duplicates. Bound antibodies were detected using alkaline phosphatase-conjugated goat anti-mouse IgM or IgG1 (SouthernBiotech, United States) diluted 1:1000. 4-nitrophenil phosphate-disodium salt (Serva, Germany) was used as substrate. IgM and IgG1 serum titers were determined by using respective isotype-specific Ig standards (SouthernBiotech, United States).

### Statistical Analysis

Results are expressed as arithmetic means ± standard deviation. Statistical analysis was performed with a two-tailed unpaired Student’s t-test or Mann-Whitney-U test as indicated, with GraphPad Prism V8 software. *p < 0.05, **p < 0.01, ***p < 0.001, ****p < 0.0001.

## Results

### Vav1 Protein Deficiency Affects Maturation of Recirculating B Cells in the Bone Marrow

**FIGURE 1:**
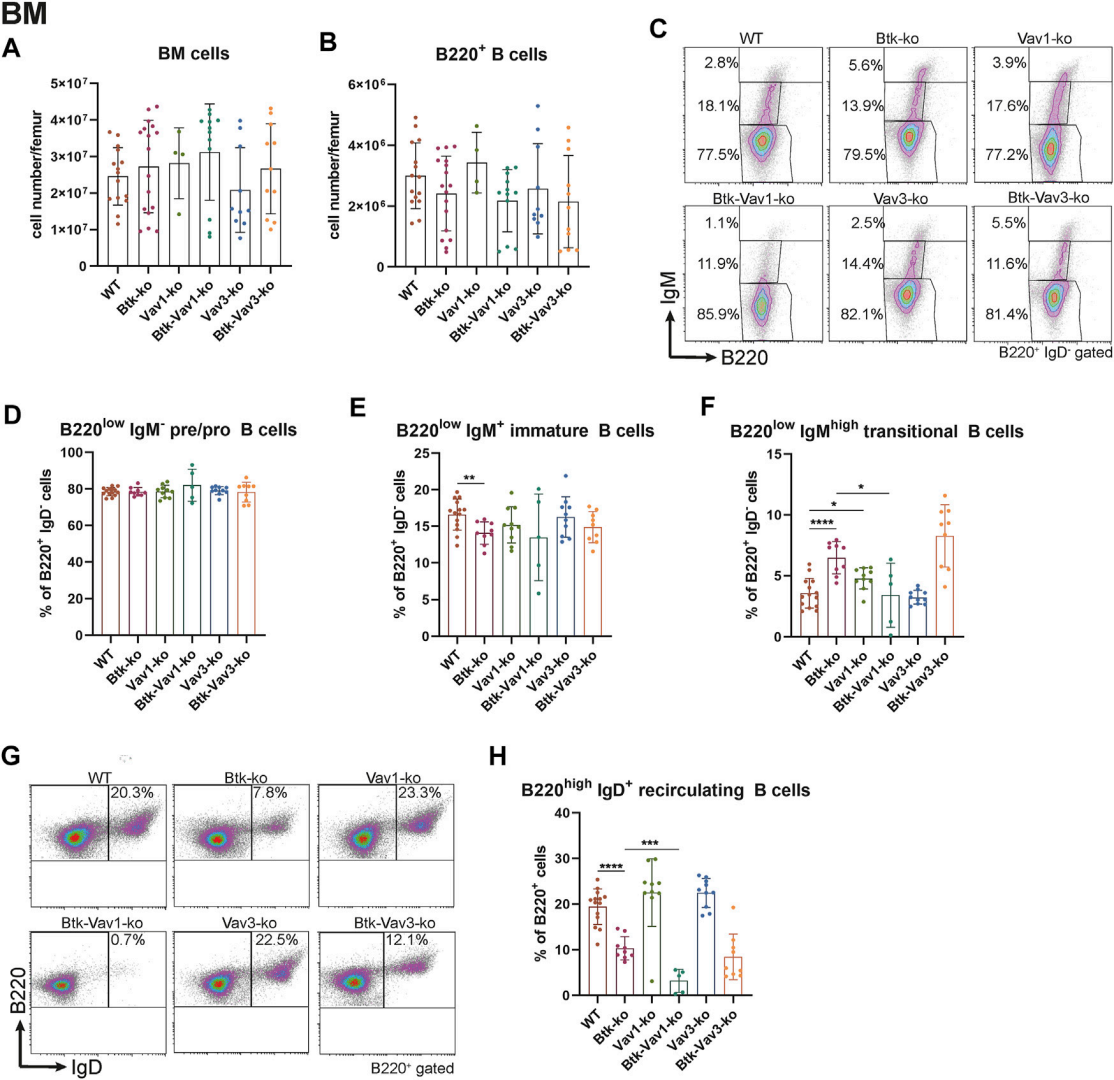
Vav1 protein deficiency affects maturation of recirculating B cells in the BM. Statistical analyses of flow cytometry representing total BM cells **(A)**, total B cells **(B)**, representative flow cytometry plots of IgM/B220 B cell subpopulations of each genotype **(C)**, statistical analyses of flow cytometry representing percentages of pro/pre B (B220^low^ IgM^−^) **(D)**, immature (B220^low^ IgM^+^) (**E**), transitional (B220^low^ IgM^high^) **(F)**, representative flow cytometry plots of recirculating B cells for each genotype **(G)** and percentages of recirculating (B220^high^ IgM^+^) **(H)** B cells. Each point represents data from a single mouse. Data in the graphs are shown as means ± SD (*n* = at least 4 mice per group). Data are merged from at least three independent experiments. *p < 0.05, and **p < 0.01. *p*-values were determined using a two-tailed Student’s t test or Mann-Whitney-U test. WT, Vav1-, Vav3-and Btk/Vav3-knockouts were analyzed in an age range from 7–15 and Btk- and Btk/Vav3-knockouts from 8–29 weeks (3 mice each were analyzed in the age range from 26–29 weeks, remaining animals were 8–14 weeks old). Gating strategy is shown in Supplementary Figure S4.

To further elucidate the role of Vav1 and Vav3 on B cell development and their possible implication in Btk-dependent signaling, we characterized different B cell subpopulations in WT, Btk-, Vav1-or Vav3-single-deficient mice in comparison with Btk/Vav1-or Btk/Vav3-double deficient animals. First, the different B cell developmental stages in the bone marrow (BM) were analyzed by flow cytometry. Total BM and total B220^+^ B cell numbers did not markedly differ between the different mouse strains (Figures 1A,B) and also the level of pro/pre B (B220^low^ IgM^−^) cells was comparable among the analyzed mouse genotypes (Figures 1C,D). As previously described, we found significantly reduced levels of immature B (B220^low^ IgM^+^) cells in Btk-deficient mice (Figures 1C,E, Supplementary Table S1). Levels of immature B cells in Vav-single deficient mice were comparable to the WT situation and a Btk/Vav-double knockout did not exacerbate Btk-related defects (Figures 1C,E, Supplementary Table S1).

Transitional B (B220^low^ IgM^high^) cell levels in the BM of Btk-knockout mice were significantly increased compared to the WT situation as described by us previously (Brunner et al., 2005) (Figures 1C,F). Vav-1 single deficiency also resulted in a modest increase of transitional B cells compared to WT controls (Figures 1C,F). Levels of recirculating B (B220^high^ IgM^+^ IgD^+^) cells were clearly reduced in Btk-deficient mice but not affected in Vav1-or Vav3-single knockouts (Figures 1G,H). Btk/Vav3-double deficient animals revealed markedly reduced numbers of recirculating B cells compared to Vav3-single knockouts probably attributable to the Btk-deficiency (Figures 1G,H). Most prominently, Btk/Vav1-double deficiency led to significantly reduced levels of recirculating B cells compared to both Btk- and Vav1-single deficient mice suggesting a direct effect of Vav1 for maturation of recirculating B cells in the BM (Figures 1G,H).

### Vav Protein Deficiency Affects B Cell Maturation in Peripheral Lymphoid Organs

**FIGURE 2:**
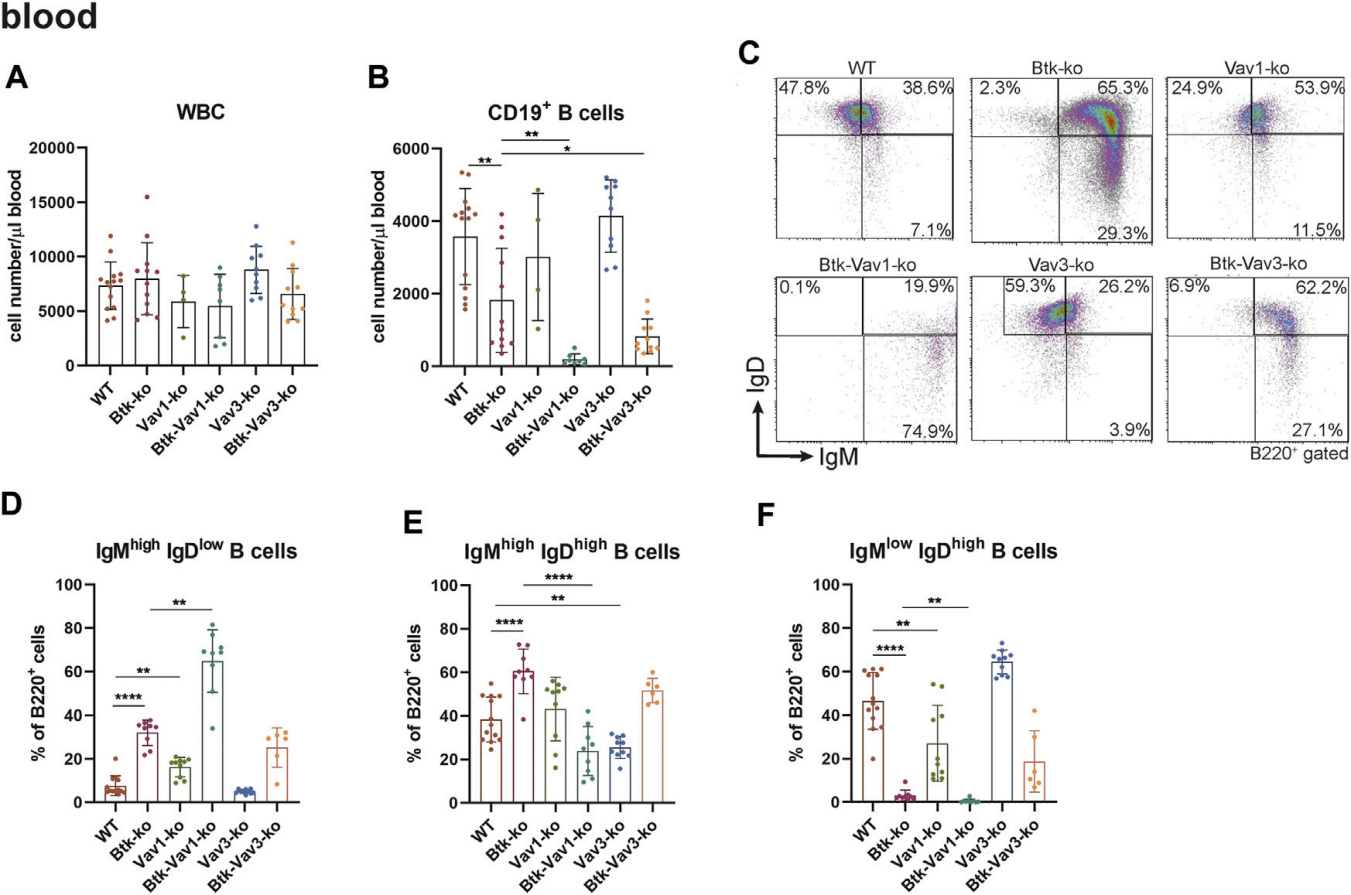
Btk/Vav double deficiency causes reduced B cell numbers in the blood. **(A)** Numbers of total WBCs measured with an Animal Blood Counter are shown. Statistical analyses of flow cytometry representing total CD19^+^ B cells **(B)**, representative flow cytometry plots of IgM/IgD B cell subpopulations in the blood for each genotype **(C)**, statistical analyses of flow cytometry representing IgM^high^ IgD^low^ **(D)**, IgM^high^ IgD^high^ **(E)** and IgM^low^ IgD^high^ **(F)** B cell percentages in the blood are shown. Each point represents data from a single mouse. Data in the graphs are shown as means ± SD (*n* = at least 4 mice per group). Data are merged from at least three independent experiments. *p < 0.05, and **p < 0.01. *p*-values were determined using a two-tailed Student’s t test or Mann-Whitney-U test. WT, Vav1-, Vav3-and Btk/Vav3-knockouts were analyzed in an age range from 7–15 and Btk- and Btk/Vav3-knockouts from 8–29 weeks (3 mice each were analyzed in the age range from 26–29 weeks, remaining animals were 8–14 weeks old). Gating strategy is shown in Supplementary Figure S5.

To further evaluate the effect of Vav1 and Vav3 on B cell development, we characterized different B cell subpopulations in the periphery of our different mouse strains representing single Btk, Vav1 or Vav3 deficiencies or combinations of Btk with either Vav1 or Vav3 deficiencies. In the blood, the number of white blood cells (WBCs) was relatively comparable between the different mouse strains (Figure 2A). Numbers of total CD19^+^ B cells in the blood was markedly reduced in Btk-deficient mice compared to the WT situation, whereas Vav1-or Vav3-single deficiency had no significant effect on CD19^+^ B cell numbers (Figure 2B). Interestingly, Btk/Vav1-and Btk/Vav3-double deficiency caused significantly reduced levels of CD19^+^ B cells compared to both Btk- and respective Vav-single-knockouts indicating an additional effect of Vav proteins on peripheral B cell development (Figure 2B). To elucidate whether the observed reduction of CD19^+^ B cells is a consequence of defective B cell maturation, B cells were further characterized for surface expression of IgM and IgD by flow cytometry. Newly from the BM emigrated B cells express high levels of surface IgM and little IgD. As B cells mature, IgM levels decrease while IgD levels increase so that mature B cells express low levels of surface IgM and high levels of IgD. Btk- and Vav1-single deficiency resulted in a significantly increased percentage of immature B cells (IgM^high^ IgD^low^) and at the same time a dramatic decrease of mature IgM^high^ IgD^low^ B cells (Figures 2C–F, Supplementary Table S2). A Btk/Vav1-double deficiency worsened Btk-related B cell maturation defects in the blood (Figures 2D,F). Levels of immature IgM^high^ IgD^low^ and mature IgM^low^ IgD^high^ B cells of Vav3-single deficient mice were comparable to the WT controls (Figures 2D,F). Collectively, these findings indicate that a single Vav deficiency seems to have an effect on B cells present in the blood circulation, while a Btk/Vav1-double deficiency exacerbates the developmental defects observed in Btk-single knockouts.

**FIGURE 3:**
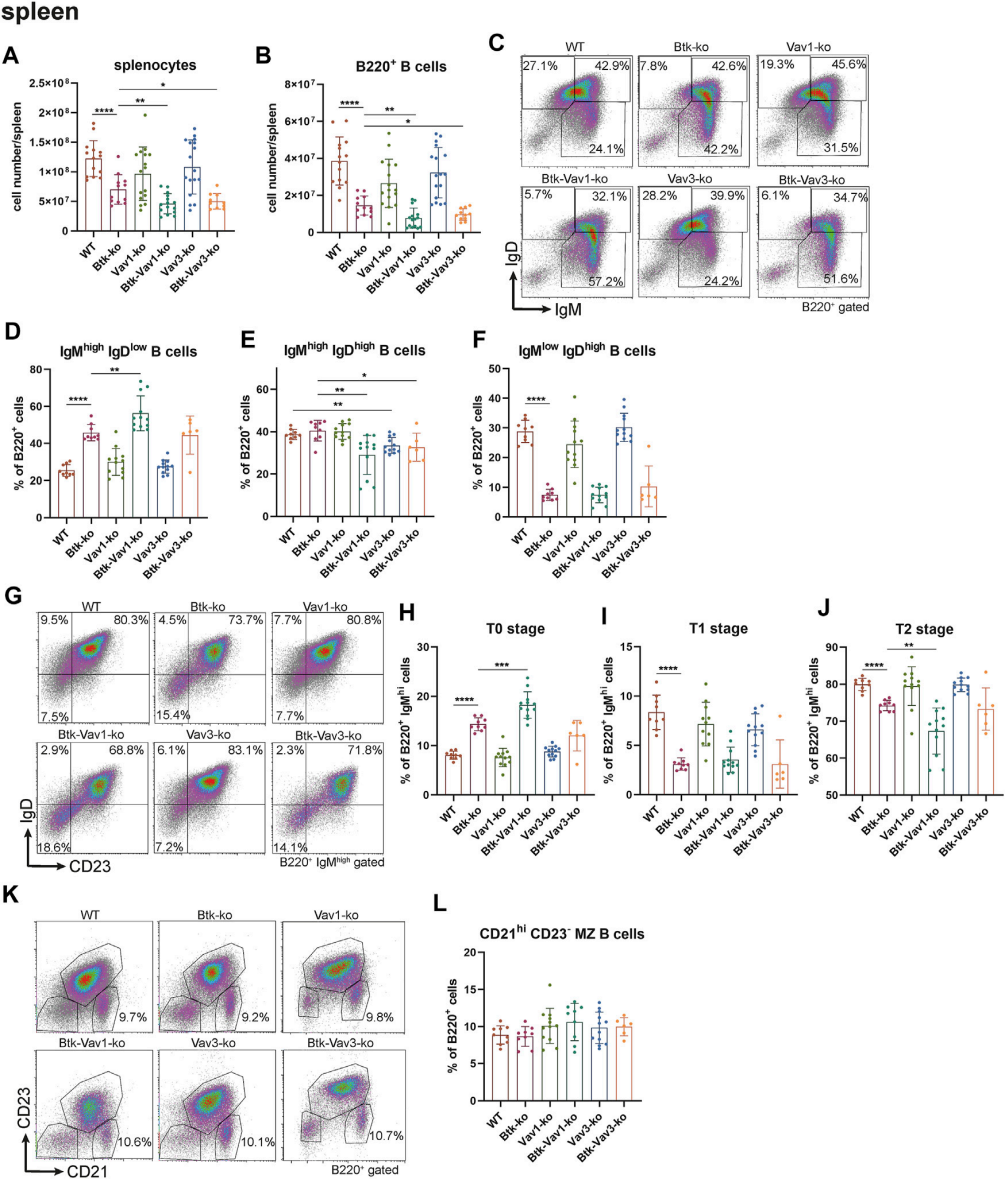
Vav protein deficiency affects B cell maturation in the spleen. Statistics of flow cytometry representing splenocytes **(A)**, total B220^+^ B cells **(B)**, representative flow cytometry plots of splenic IgM/IgD B cell subpopulations of each genotype **(C)**, statistics of flow cytometry representing percentage of IgM^high^ IgD^low^ **(D)**, IgM^high^ IgD^high^ **(E)** and IgM^low^ IgD^high^ **(F)** B cells in the spleen. Representative flow cytometry plots of splenic T0, T1 and T2 stage B cell subpopulations of each genotype **(G)**, statistics of flow cytometry representing percentage of T0 stage **(H)**, T1 stage **(I)** and T2 stage **(J)** B cells. Representative flow cytometry plots **(K)** and statistics **(L)** of MZ B cells. Each point represents data from a single mouse. Data in the graphs are shown as means ± SD (n = at least 6 mice per group). Data are merged from at least three independent experiments. *p < 0.05, **p < 0.01, ***p < 0.001, and ****p < 0.0001. *p*-values were determined using a two-tailed Student’s t test or Mann-Whitney-U test. Mice were analyzed in an age range from 10–16 weeks. Gating strategy is shown in Supplementary Figure S6.

Since flow cytometric analyses of the blood suggested an effect of Vav protein deficiency on B cell maturation, we wondered whether Vav proteins are also required for sufficient development of the spleen. Therefore, we further investigated the splenic cell population by flow cytometry. Here, both the number of splenocytes and that of total B cells were considerably decreased in Btk-deficient mice (Figures 3A,B). Numbers of splenocytes and total B cells in Vav1-and Vav3-single deficient mice were comparable to the WT situation (Figures 3A,B, Supplementary Table S3). However, Btk/Vav1-and Btk/Vav3-double deficient animals revealed significantly reduced splenocytes and B cells compared to Btk-single deficient mice (Figures 3A,B). These data suggest a worsening of B cell developmental defects described for Btk-knockout mice in animals with additional Vav1 or Vav3 deficiency. Since splenocytes and B cell numbers were reduced upon additional Vav protein deficiency, we further investigated different B cell developmental stages in the spleen of our knockout mice by flow cytometry. In line with our results obtained by flow cytometric analyses of the blood, we observed a significant increase in the percentage of immature IgM^high^ IgD^low^ B cells upon Btk-single and Btk/Vav1-or Btk/Vav3-double deficiency (Figures 3C,D, Supplementary Table S2). The Btk/Vav1-double deficiency even significantly potentiated the developmental defect observed in Btk-single deficient mice (Figure 3D). In contrast, the percentage of IgM^high^ IgD^high^ B cells was significantly reduced in both Btk/Vav1-and Btk/Vav3-double deficient mice compared to a Btk-single deficiency (Figure 2E). The percentage as well as the cell number of mature IgM^low^ IgD^high^ B cells were dramatically reduced in Btk-single as well as Btk/Vav1-and Btk/Vav3-double knockouts (Figures 3C,F, Supplementary Figure S1C). To get further insights into the B cell development of our different mouse strains, we next assessed the levels of transitional T0, T1 and T2 B cells according to Henderson et al. (2010). Transitional B cells represent B cells at an intermediate stage in their development between immature B cells from the BM and mature splenic B cells. By assessing B cell percentages of T0/T1/T2 stages we could provide further evidence for B cell developmental defects in Btk/Vav1-and Btk/Vav3-double deficient animals in line with our data obtained by analyzing surface expression of IgM/IgD. Btk-single as well as both double knockouts featured a significant increase in the proportion of T0 (IgM^high^ IgD^−^ CD23^−^) B cells compared to the WT situation and the respective Vav-single knockouts (Figures 3G,H). The Btk/Vav1-double deficiency again significantly potentiated the defect observed in Btk-single knockouts (Figure 3H). The percentage of T1 (IgM^high^ IgD^+^ CD23^−^) and T2 (IgM^high^ IgD^+^ CD23^+^) B cells was markedly reduced in Btk-single and Btk/Vav1-and Btk/Vav3-double knockouts (Figures 3G,I,J). The reduction of T2 levels was more prominent in Btk/Vav1-double knockouts compared to Btk-single deficient mice (Figure 3J, Supplementary Figure S1F). Next, we analyzed the levels of Marginal Zone B (MZ B) cells to further evaluate whether our observed differences in the IgM/IgD profile could be due to defective MZ B cell development, since they are also IgM^high^ expressing cells. Therby, we could not detect differences in the levels of MZ B cells between the different mouse genotypes (Figures 3K,L). In summary, these results provide evidence for B cell developmental defects in Btk/Vav1-and Btk/Vav3-double deficient mice and suggest a requirement especially for Vav1 for efficient B cell maturation in the periphery.

### Vav Protein Deficiency Causes Disturbed Microarchitecture of the Spleen

**FIGURE 4:**
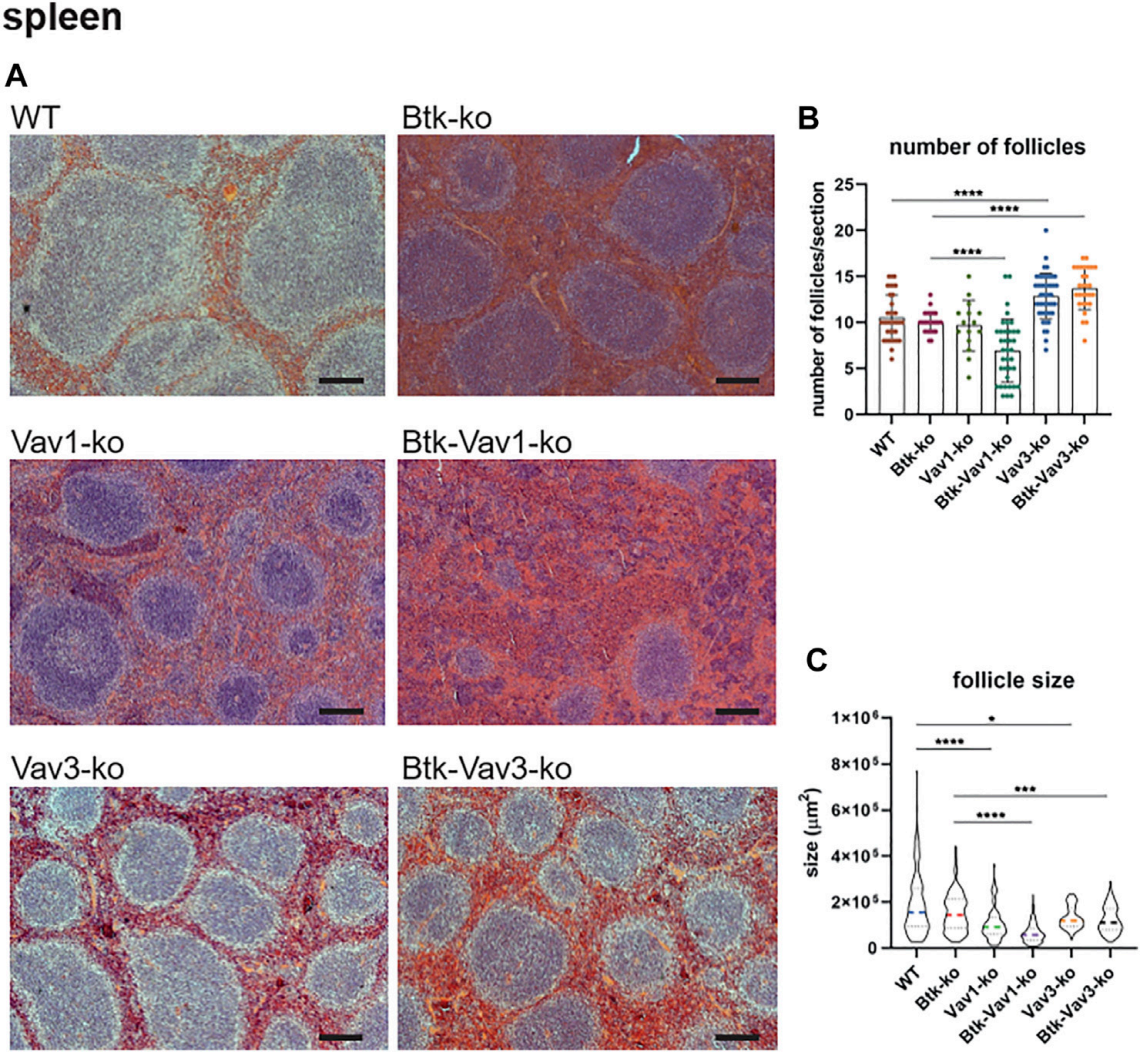
Vav protein deficiency causes disturbed splenic microarchitecture. **(A)** Representative HE-stainings of splenic sections Scale bar indicates 200 µm. **(B, C)** Statistical analyses showing number of follicles **(B)** and follicle size **(C)** determined within a certain section. Three sections were analyzed per animal. Each point represents one analyzed image section. Data in the graphs are shown as means ± SD. Spleens of WT (*n* = 12), Btk-ko (*n* = 9), Vav1-ko (*n* = 5), Btk/Vav1-ko (*n* = 13), Vav3-ko (*n* = 15) and Btk/Vav3-ko (*n* = 9) animals were stained and analyzed. Data are merged from at least three independent experiments. *p < 0.05, **p < 0.01, ***p < 0.001, and ****p < 0.0001. *p*-values were determined using a two-tailed Student’s t test or Mann-Whitney-U test. Mice were analyzed in an age range from 9–13 weeks

Since flow cytometric data suggested an effect of Vav protein deficiency on splenocytes and B cell maturation, we wondered whether the splenic microarchitecture was also affected in these mice. Consequently, we assessed the splenic microarchitecture of our different mouse strains by HE staining (Figure 4A). Thereby, number and size of follicles in Btk-single deficient mice was comparable to the WT situation (Figures 4B,C). Vav3-single deficient mice were characterized by increased numbers of follicles that were at the same time significantly smaller in size compared to the WT situation (Figures 4B,C). Btk/Vav3-double deficiency also revealed markedly increased follicle numbers that were significantly smaller in size in comparison to Btk-single deficiency (Figures 4B,C). However, Btk/Vav3-double deficiency had no additional effect in comparison to Vav3-single deficiency. Vav1-single deficient mice showed a distinct reduction of follicle size, whereas the follicle number was unaffected (Figures 4B,C). Btk/Vav1-double deficient mice featured the most striking disturbed splenic microarchitecture, since they displayed dramatically reduced numbers and size of follicles compared to both Btk- and Vav1-single deficient mice (Figures 4B,C). Collectively, these data indicate that both Vav-1 and Vav3-single deficiency causes disturbed splenic microarchitecture and that additional deficiency of Vav proteins potentiates the defects observed in Btk-single deficient mice.

### Vav Protein Deficiency Affects B Cell Proliferation and Exacerbates Ca^2+^ Mobilization Defects

**FIGURE 5:**
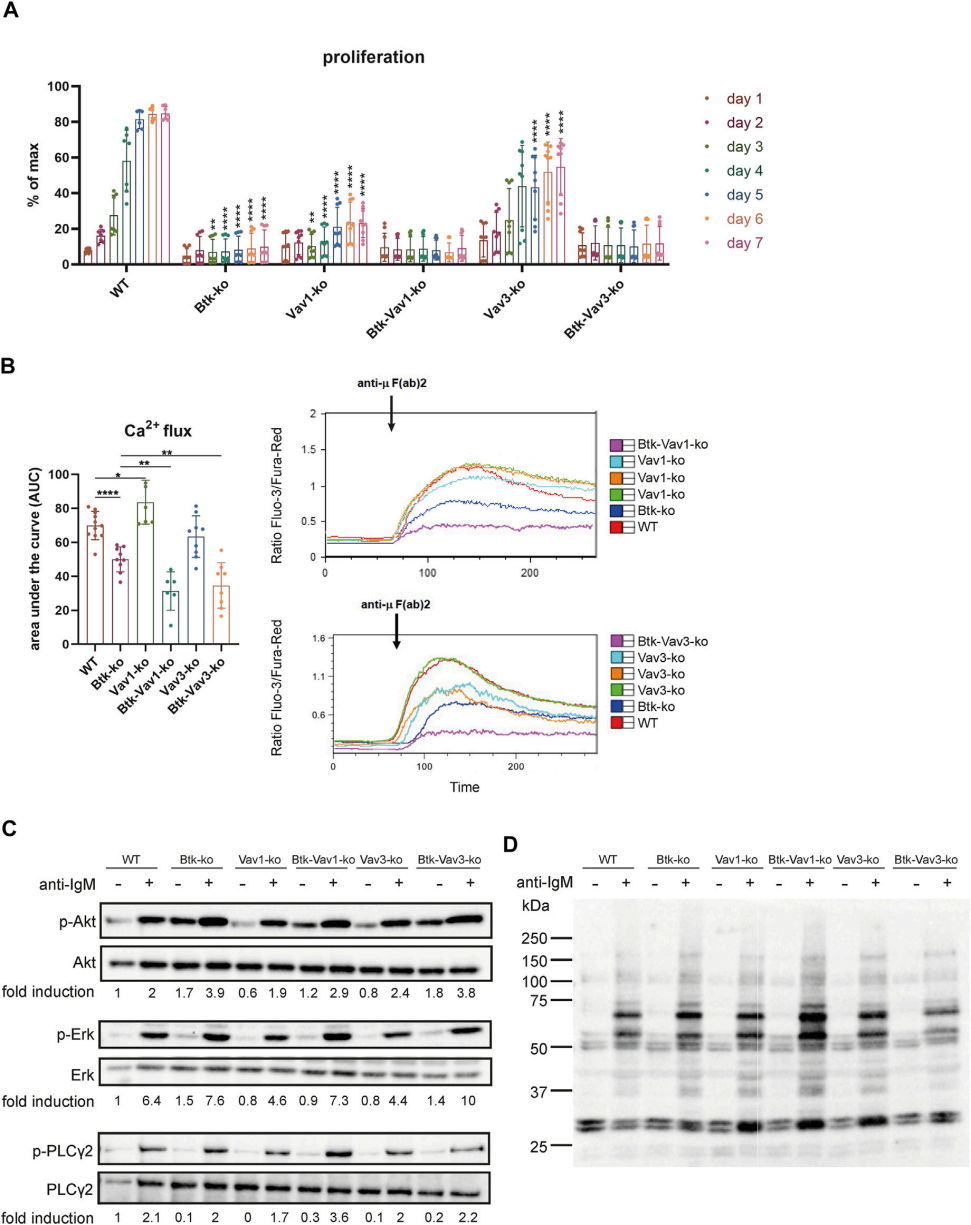
Vav-protein deficiency affects B cell proliferation and exacerbates Ca2+ mobilization defects. **(A)** Statistical analysis of flow cytometry data showing CFSE dilution of B cells. Splenocytes were labeled with CFSE and proliferation of B220^+^ B cells was monitored over 7 days. Asterisks indicate significances compared to WT control animals. **(B)** Statistics of Ca^2+^ influx showing the area under the curve upon anti-µ F (ab) 2 stimulation (left). Representative plots depicting Ca^2+^ influx over time after anti-µ F (ab) 2 stimulation are shown (right). Each point represents data from a single mouse. Data in the graphs are shown as means ± SD (n = at least 6 mice per group). Data are merged from three independent experiments. *p < 0.05, **p < 0.01, ***p < 0.001, and **** < *p* 0.0001. *p*-values were determined using a two-tailed Student’s t test or Mann-Whitney-U test. **(C)** Naïve B cells were isolated from respective mouse strains and stimulated with anti-µ F (ab) 2 for 3 min. Cells were lysed and phosphorylated forms of Akt (S473), Erk (Y204) and PLCγ2 (Y1217) as well as phospho-tyrosine were assessed by western blot. Non-phosphorylated proteins were used as loading controls. Representative image of 2 independent experiments. Mice were analyzed in an age range from 7–14 weeks

To evaluate the effect of Vav protein deficiency on B cell function, we monitored proliferation of CFSE stained cells isolated from spleens of our different mouse strains over 7 days. Upon BCR cross-linking by stimulation with anti-µ F (ab)2, B cells of Btk- and Vav1-single deficient mice revealed almost no proliferation (Figure 5A). On the other hand, B cells of Vav3-single deficient mice showed an increase in proliferation over time resulting in 50% of B cells proliferating on day 7. However, the capacity to proliferate was still markedly reduced in Vav3-single knockouts compared to the WT situation, especially from day 5 to day 7 (Figure 5A). B cells of both Btk/Vav1-and Btk/Vav3 double deficient animals were virtually unable to proliferate. These findings indicate that in the absence of Vav proteins, especially Vav1, B cells are impaired in their proliferative capacity (Figure 5A).

Since each of Btk, Vav1 and Vav3 itself has already been shown to be involved in Ca^2+^ mobilization upon BCR activation, we wondered how Btk/Vav1-or Btk/Vav3-double deficiency impacts the ability to increase intracellular Ca^2+^ levels. As expected, Btk-single deficient mice were characterized by a dramatic reduction of Ca^2+^ influx compared to the WT situation (Figure 5B). In line with previous studies, Vav1-single deficiency resulted in increased Ca2^+^ flux compared to WT controls (Figure 5B). Strikingly, Btk/Vav1-as well as Btk/Vav3-double deficiency significantly make the Ca^2+^ signaling defects observed in Btk-single knockout mice more severe (Figure 5B). Thus, both Vav proteins appear to be required for Ca^2+^ mobilization upon BCR activation.

To get further insights whether Vav proteins contribute to B cell development and activation through signaling in a Btk-dependent or independent manner, we analyzed anti-IgM induced phosphorylation of Akt, Erk and PLCγ2 by western blot. Therefore, the phosphorylation level of these proteins was compared between unstimulated and stimulated B cells isolated from our different mouse strains. The extent of phosphorylation following anti-IgM stimulation was quantified by densitometry and was not significantly affected in B cells lacking any combination of Btk and Vav proteins (Figure 5C, Supplementary Figure S2). Stimulated B cells from all knockout strains revealed an increased phosphorylation level compared to respective unstimulated B cells. Quantification of phosphorylated Akt, Erk and PLC γ2 revealed no significant differences between the different knockout mice (Supplementary Figure S2E,F). We also could not detect serious differences in the intensity of total tyrosine phosphorylation between the different mouse genotypes (Figure 5D, Supplementary Figure S2).

### Vav1 Deficiency Impairs Efficient Germinal Center Formation and Subsequent Production of Secondary Immunoglobulins

**FIGURE 6:**
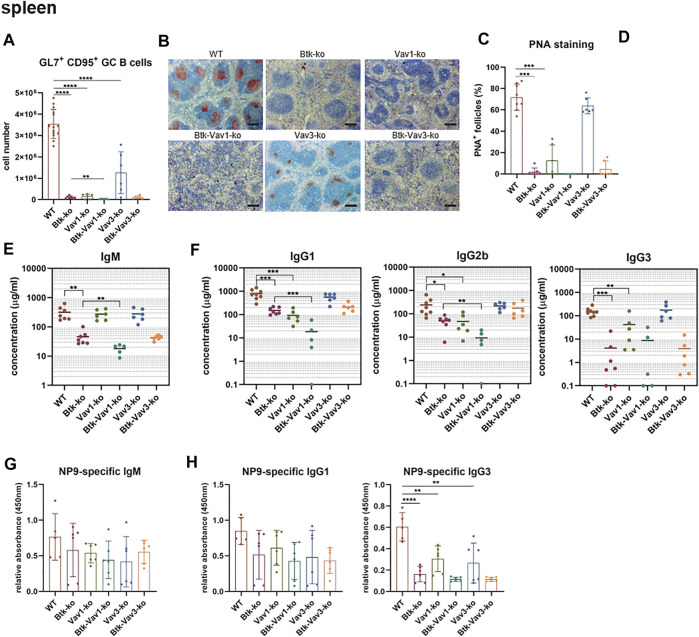
Vav1 deficiency impairs efficient GC formation and immunoglobulin production. Mice were immunized with SRBCs and GC formation was assessed by flow cytometry and immunohistochemistry and immunoglobulin serum titers were determined by ELISA. **(A)** Statistics of flow cytometric analyses of GC B (GL7^+^ CD95^+^) cell numbers in the spleen are shown. **(B)** Representative immunohistochemical staining of PNA^+^ GCs for each genotype and are shown. Scale bars indicate 200 µm **(C)** Statistical analyses showing ratio of PNA^+^ GCs/total follicles stained by immunohistochemistry. **(D)** Statistical analysis showing GC size of WT (*n* = 10) and Vav3-single knockouts (*n* = 5). Size of GCs of three image sections for each animal was measured. The mean of all three analyzed image sections for each mouse is depicted. **(E)** Statistical representation of IgM serum concentrations (µg/ml) determined relative to a standard IgM. **(F)** Statistical representation of IgG1, IgG2b and IgG3 serum concentrations (µg/ml) determined relative to corresponding standard antibodies. **(G,H)** Mice were immunized with NP-KLH to determine levels of antigen-specific IgM **(G)** as well as IgG1 and IgG3 **(H)**. Each point represents data from a single mouse. Data in the graphs are shown as means ± SD (n = at least 5 mice per group). Data are merged from two independent experiments. *p < 0.05, **p < 0.01, and ***p < 0.001. *p*-values were determined using a two-tailed Student’s t test or Mann-Whitney-U test. Mice were analyzed in an age range from 7–13 weeks. Gating strategy is shown in Supplementary Figure S7.

To examine the effect of Vav protein deficiency on Germinal Center (GC) formation, we immunized mice with sheep red blood cells (SRBCs). Flow cytometric analyses revealed that GL7^+^ CD95^+^ GC B cells are almost absent in Btk- and Vav1-single as well as in Btk/Vav1-double deficient mice (Figure 6A). Interestingly, Btk/Vav1-double deficient mice were characterized by significantly reduced GC B cell numbers compared to Btk-single knockouts (Figure 6A). GC B cells were also significantly reduced in Vav3-single deficient mice compared to the WT situation (Figure 6A). In Btk/Vav3-double deficient animals GC B cells were almost absent probably as a consequence of Btk deficiency, since this decrease was not significant compared to Btk-single knockouts (Figure 6A). Histological analyses of PNA^+^ GCs numbers confirmed results obtained by flow cytometry (Figures 6B,C). In addition, histological analyses revealed that the size of GCs found in Vav3-single knockouts is markedly reduced compared to WT controls (Figures 6B,D). These findings suggest a requirement of both Vav proteins for efficient GC formation. Since an efficient GC reaction results in the production of class-switched secondary immunoglobulins, we assessed IgM and class-switched IgG immunoglobulins levels in serum of our single and double knockout mouse strains by ELISA. IgM serum levels were extremely reduced in Btk-single as well as in both Btk/Vav1-and Btk/Vav3-double deficient mice, whereas IgM levels in both Vav-single knockouts were comparable to the WT situation (Figure 6E). Strikingly, Btk/Vav1-double deficiency resulted in a significant reduction of IgM serum levels compared to Btk-single deficient animals (Figure 6E). Moreover, class-switched IgG1, IgG2b and IgG3 immunoglobulins were dramatically reduced in Btk- and Vav1-single as well as in Btk/Vav1-double deficient mice (Figure 6F). Here again, Btk-Vav1-double deficient mice showed significantly reduced IgG1 and IgG2b levels compared to Btk-single knockouts (Figure 6F). Class-switched IgG levels in Vav3-single knockout mice were unaffected. A Btk/Vav3-double deficiency did not potentiate the defects in immunoglobulin production observed in Btk-single knockouts (Figure 6F). To asses antigen-specific antibody responses, we immunized mice with NP-KLH. Levels of NP-specific IgM and IgG1 immunoglobulins were not significantly altered between the analyzed mouse strains (Figures 6G,H). However, NP-specific IgG1 was slightly reduced in Btk-single and Btk/Vav1 and Btk/Vav3-double deficient mice compared to the WT situation. (Figure 6H). NP-specific IgG3 immunoglobulins were significantly reduced in Btk-as well as Vav1-and Vav3-single knockouts compared to WT controls (Figure 6H). Btk/Vav1 and Btk/Vav3-double deficiency also resulted in a distinct reduction of antigen-specific IgG3 compared to WT and respective Vav-single knockouts (Figure 6H). Collectively, these data indicate a requirement of Vav1 protein for efficient GC formation and function.

### Vav Protein Deficiency Impairs B Cell Differentiation Into ASCs *in vitro*

**FIGURE 7:**
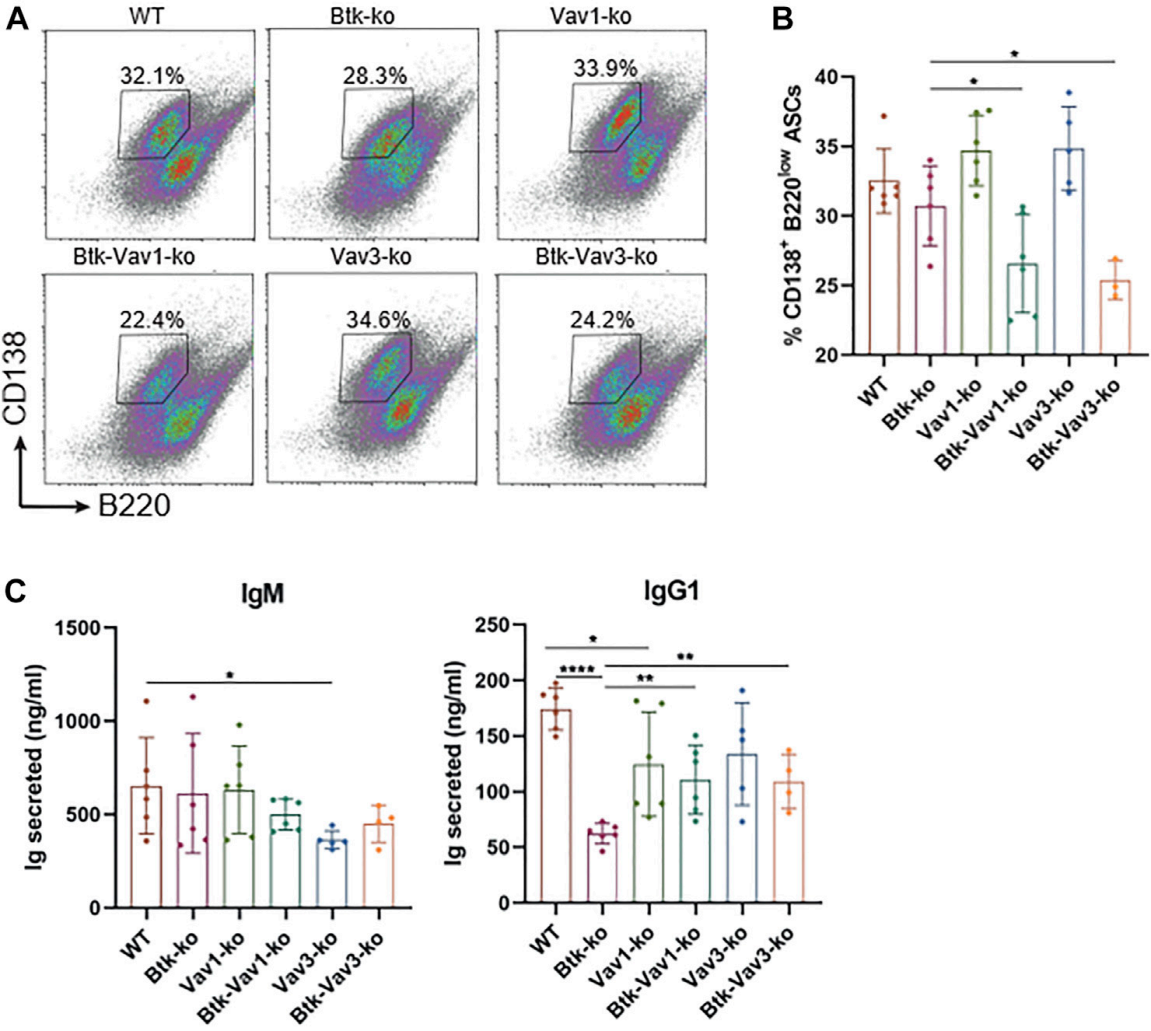
Vav protein deficiency impairs B cell differentiation into ASCs *in vitro*. Naïve B cells were isolated from respective mouse strains and cultured in the presence of CD40 and IL4 for 7 days. **(A. B)** On day 4, cells were collected and analyzed for the presence of CD138^+^ B220^low^ ASCs by flow cytometry. Representative flow cytometry plots for each genotype (*A*) and statistical analysis **(B)** are shown. **(C)** On day 7, culture supernatants were collected and secreted amounts of IgM and IgG1 are shown. Data are merged from three independent experiments. *p < 0.05, **p < 0.01, and ***p < 0.001. *p*-values were determined using a two-tailed Student’s t test or Mann-Whitney-U test. Mice were analyzed in an age range from 10–13 weeks

Since Vav protein expression is not restricted to B lymphocytes and impaired responses to thymus-dependent antigens in Vav1-deficient animals have been reported as a consequence of defective T cell help by Vav-deficient T cells (Bachmann et al., 1999; Gulbranson-Judge et al., 1999), we tested whether there is also a Vav-mediated B cell intrinsic defect contributing to impaired GC formation and function. Therefore, we isolated B cells from our mouse strains and differentiated them into antibody secreting cells (ASCs) in the presence of CD40L and IL4 *in vitro*. On day 4, percentage of B cells carrying the ASC surface markers CD138^high^ B220^low^ addressed by flow cytometry was significantly reduced in both Btk/Vav1-and Btk/Vav3-double knockouts compared to Btk-single deficient mice (Figures 7A,B). However, percentage of ASCs in Vav1-and Vav3-single knockouts was comparable to the WT situation (Figures 7A,B). On day 7, immunoglobulins secreted into the medium were measured by ELISA. IgM levels were comparable between the different knockouts, except for Vav3-single deficient mice, which revealed significantly reduced levels compared to WT controls (Figure 7C). Both Btk- and Vav1-single deficient animals featured significantly reduced levels of secreted IgG1 compared to WT controls (Figure 7C). However, the reduction of IgG1 in both double-knockouts was not as prominent as in Btk-single knockout mice (Figure 7C). ASC differentiation is known to be associated with the expression of transcription factors like *prdm1* and *irf4* and the suppression of *pax5* and *bcl6*. Therefore, we checked expression levels of these transcription factors on day 7 of culture by qRT-PCR. However, no significant differences in expression levels of analyzed transcription factors could be observed between the different mouse strains. There was a slight reduction in the expression of *prdm1* and *irf4* in both double-knockouts compared to the WT situation and respective Vav-single knockouts (Supplementary Figure S2). On the other hand, there was a slight increase in *pax5* expression level in Btk/Vav1-double and Vav3-single deficient mice compared to WT and Btk-knockout mice (Supplementary Figure S2). These results suggest that the impaired immune responses observed upon Vav1 or Vav3 protein ablation are the result of a contribution of both B but also T cells.

## Discussion

Since their discovery, Vav proteins were mainly studied in T cells and their role, particularly that of Vav3, for B cell maturation and BCR signaling is incompletely understood. The development and survival of B cells is crucially dependent on BCR signaling (Lam et al., 1997; Levine et al., 2000). Upon BCR engagement, proteins including Btk, Vav, BLNK, PLC-γ2 and PI3K interact to form the signalosome thereby regulating the level of intracellular calcium and subsequent downstream events (DeFranco, 2001; Turner, 2002; Turner and Billadeau, 2002; Löber et al., 2020). Vav proteins become phosphorylated and activated upon BCR cross-linking and can subsequently regulate PI3K activity ascribing them a central role during BCR signaling (Bustelo, 2000; Turner, 2002). By now, predominantly a deficiency of Vav proteins alone or in combination with other Vav protein family members have been studied. Since Vav proteins and Btk are components of one signaling complex termed the signalosome responsible for BCR-mediated signal transduction and the phenotype of Vav mutant mice resembles that of Btk-deficient animals it was postulated that these proteins lie in the same signal transduction chain. However, this assumption has never been examined in detail. To do this, we investigated the effects of a simultaneous deficiency of Vav1 or Vav3 and Btk. The analysis of Vav2-deficient animals was excluded from this study, since Vav2-deficient B cells neither showed severe Ca^2+^-flux deficits nor obvious B developmental defects (Doody et al., 2001; Tedford et al., 2001; Löber et al., 2020). Our analyses revealed that Vav molecules are involved in Btk-dependent and -independent signaling pathways important for B cell development and function.

In line with previous findings (Khan et al., 1995; Hendriks et al., 1996), we observed a reduction of immature and recirculating B cells in the BM of Btk-deficient mice. Besides, we observe an increased level of transitional B cells in the BM of Btk-knockout mice compared to WT. Transitional B cells represent the link between BM immature and peripheral mature B cells. Transitional B cells retain key phenotypic characteristics of their BM counterparts when they leave the BM and enter the peripheral circulation (Chung et al., 2003). To further characterize the development from the immature B cell stage on, Khan et al. characterized the expression of IgM^+^ B220^int^ and Hendriks et al. evaluated IgM/IgD surface expression. In this study, we characterized transitional B cells of the BM by their expression of IgM^high^ B220^low^ according to Hess et al. (2001). It is well recognized that there is a defect in the transition from pre/pro B cells to immature B cells in the BM as well as an accumulation of transitional B cells in the periphery due to an arrest from the IgM^high^ IgD^low^ to the IgM^low^ IgD^high^ stage in Btk-knockout mice (Khan et al., 1995; Hendriks et al., 1996). It might also be possible that Btk-deficient transitional B cells have defects to exit the BM, which might additionally account for reduced B cell numbers in the periphery and would explain the observed increase in the number of transitional B cells in our study.

In the periphery, we observed a block of BTK-deficient peripheral B cells at the T0 stage, with an accumulation of B cells at T0 and reduced numbers of T1 and T2 Btk-deficient B cells. This B cell maturation defect was even more pronounced in Btk/Vav1-double deficient animals. In contrast, Allman et al. (2001) previously revealed a mild reduction of T1 and T2 cells, but a dramatic decrease in Btk-deficient T3 B cells in the absence of Btk. In our analysis we adhered to the gating strategy used by Henderson et al., (Henderson et al., 2010), allowing to distinguish T0, T1 and T2 B cell developmental stages according to the expression of IgM, IgD and CD23. In contrast, Allman et al. (2001) used a different gating strategy in order to identify peripheral B cell populations T1, T2 and T3. In conclusion, the here described defects caused by an additional Vav1 deficiency suggest that Btk and Vav1 are involved in different signaling pathways essential for B cell development. Moreover, these findings indicate that a Vav1-single deficiency might be compensated by Btk-dependent signaling events, explaining the normal B cell development observed in Vav1-single deficient mice. Previously it was shown that Vav2 can compensate for the lack of Vav1 in signaling pathways that promote B cell maturation (Doody et al., 2001; Tedford et al., 2001). Moreover, since Vav3 can compensate for the loss of Vav1 in T cells and Vav1/Vav3-double deficiency leads to reduced B cell numbers (Fujikawa et al., 2003), it is likely that Vav3 might also compensate for the absence of Vav1 during B cell maturation. Unlike the limited impact of Vav1-single deficiency on single cell level obtained by flow cytometry, it caused an altered splenic microarchitecture. Again, this defect was worsened by a Btk/Vav1-double deficiency. These findings suggest that Vav1 itself has effects on the splenic microarchitecture in a Btk-independent way and that these defects are further exacerbated by an additional Btk deficiency. Vav3-single deficiency revealed no major effect on B cell maturation in the BM or the periphery and defects observed in Btk/Vav3-double deficient mice were comparable to Btk-single knockouts. In total, these findings suggest that Btk and Vav3 are rather involved in the same signaling pathways required for B cell maturation.

Besides B cell maturation defects, we also report defective B cell proliferation upon Vav protein ablation, suggesting a requirement of Vav proteins downstream of BCR signaling. Calcium fluxes are involved in the regulation of both B cell maturation and proliferation upon antigen-receptor engagement. Consequently, we wondered whether defective Ca^2+^ fluxes could account for the observed B cell maturation and functional defects in Vav1-and Vav3-knockouts. In line with results observed previously (Doody et al., 2001; Tedford et al., 2001; Vigorito et al., 2004), Vav1-single deficiency did not result in impaired Ca^2+^ release instead it rather increased Ca^2+^ flux. In Vav3-deficient B cells the αIgM-induced Ca^2+^ flux was comparable or just slightly reduced to that of WT B cells. However, both Btk/Vav1-as well as Btk/Vav3-double deficiency made Ca^2+^ signaling defects observed in Btk-single knockout mice more severe. In total, these findings suggest that Btk and Vav proteins regulate Ca^2+^ flux independently of each other. Vav proteins seem to regulate Ca^2+^ mobilization in several ways. It is thought that Vav controls Ca^2+^ flux by regulating PLC-γ (Manetz et al., 2001; Inabe et al., 2002). It has been shown that Vav is required for the phosphorylation of the PLC-γ activation site (Reynolds et al., 2002). Thus, the failure to activate PLC-γ might be due to defective activation of Tec family protein kinases like Btk in B cells, since BCR-stimulated Btk phosphorylation is defective in the absence of Vav (Inabe et al., 2002). However, we could not detect impaired phosphorylation of PLCγ2 in our mouse lines. In addition, there is evidence that Vav is an upstream regulator of PI3K (Manetz et al., 2001; Reynolds et al., 2002). The ability of Vav proteins to activate PI3K seems to be Rac-dependent, since BCR-mediated PI3K activation in Vav3-deficient B cells could be restored by WT Vav3, but not a GEF-deficient Vav3 (Inabe et al., 2002). Together, these studies indicate a GEF-dependent role for Vav in PI3K activation. Moreover, there is evidence indicating that the nature of the antigen determines the contribution of Vav to PI3K activation in B cells. Data of Vigorito et al. show that anti-IgM activated PI3K is independently of Vav, whereas Ca^2+^ flux upon membrane immunoglobulin/CD19 co-ligation was entirely dependent on both PI3K and Vav (Vigorito et al., 2004), which could explain that we could not observe differences in phosphorylation level of tyrosine upon stimulation with anti-IgM. Our data suggests that for precise BCR-mediated signaling events the individual role of Vav-protein family members in relation to Btk should be assessed.

In line with previous findings (Khan et al., 1995), we observed an impaired primary antibody response upon immunization with NP-KLH in Btk-deficient mice. Similarly, we also report diminished levels of serum IgM and IgG3. Since Btk-deficient mice lack B1 cells, which can produce IgM and IgG3 in response to thymus-independent antigens, the absence of these immunoglobulins in Btk-knockout mice was suggested to be a consequence of missing B1 cells. However, secretion of IgG3 by B1 cells has been studied primarily as a T-independent antibody formed early during an immune response (Savage et al., 2017). In this study we also detected markedly reduced numbers of IgG3 upon immunization with the thymus-dependent antigens NP-KLH or SRBCs. The decrease of antigen-specific IgG3 levels suggests that there is also an impaired GC function in Btk-deficient mice and that the reduced IgG3 levels are not only a consequence of reduced B1 cells. The thymus-dependent antigen-response in Vav1-single as well as in Btk/Vav1-double knockouts was severely impaired. Comparing with Btk-single knockout mice, impaired immune responses were even more pronounced in Btk/Vav1-double deficient animals, supporting the assumption that Btk and Vav1 are involved in different signaling pathways. Vav3-deficient animals on the other hand also featured significantly reduced levels of GC B cells, but the defect was less severe than in Btk- or Vav1-knockout animals. The histological analyses of Vav3-deficient mice revealed that their GCs were much smaller in size explaining the observed reduced GC B cell numbers assessed by flow cytometry. Vav3-single knockout mice also featured an impaired antigen-specific immune response. Previous studies revealed that the impaired responses to thymus-dependent antigens in Vav1-deficient animals are probably due to defective T cell help by Vav-deficient T cells, since this defect can be restored in the presence of normal T cells (Bachmann et al., 1999; Gulbranson-Judge et al., 1999). Consequently, we tested whether there is also a Vav-mediated B cell intrinsic defect contributing to impaired GC formation and function. However, results of our *in vitro* ASC differentiation cannot provide a definite answer to this question. Flow cytometric analysis revealed that a Btk/Vav-double deficiency worsens the reduced capacity of Btk-single deficient B cells to differentiate into ASCs. On the other hand, when assessing the amount of secreted immunoglobulins or the expression of key ASC transcription factors on mRNA level this phenotype was ameliorated. Altogether, our findings suggest that a contribution of both B and T cells is responsible for the phenotype observed in the here described Btk/Vav-double deficient mice.

Collectively, our analyses revealed novel roles of Vav1 and Vav3 in B cell development and function. Moreover, our data suggests Btk-dependent and–independent roles for Vav1 and Vav3 in B cell signaling. Whereas Vav1 and Btk seem to be involved in different signaling pathways, Vav3 and Btk seem to lie in the same signal transduction chain both during B cell maturation and after antigen encounter. Thus, the BCR-induced signalosome is a complex multi-protein structure, in which different Vav-family members can coordinate BCR-mediated signaling in different ways - necessary for Btk-dependent as well as independent signal transduction. To fully complete this complex picture, it is important to note that the here described phenotype of a Btk/Vav-double deficiency is probably related to a contribution of both B and T cells.

## Data Availability

The original contributions presented in the study are included in the article/Supplementary Material, further inquiries can be directed to the corresponding author.
