# Supplementary material for: Differential Requirement of Vav Proteins for Btk-dependent and –Independent Signaling During B Cell Development
Source: Front Cell Dev Biol. 2022 Feb 23;10:654181. doi: 10.3389/fcell.2022.654181 (PMC8904969; doi:10.3389/fcell.2022.654181)
Supplement: Supplementary file 1 [file DataSheet1.PDF]

Spleen

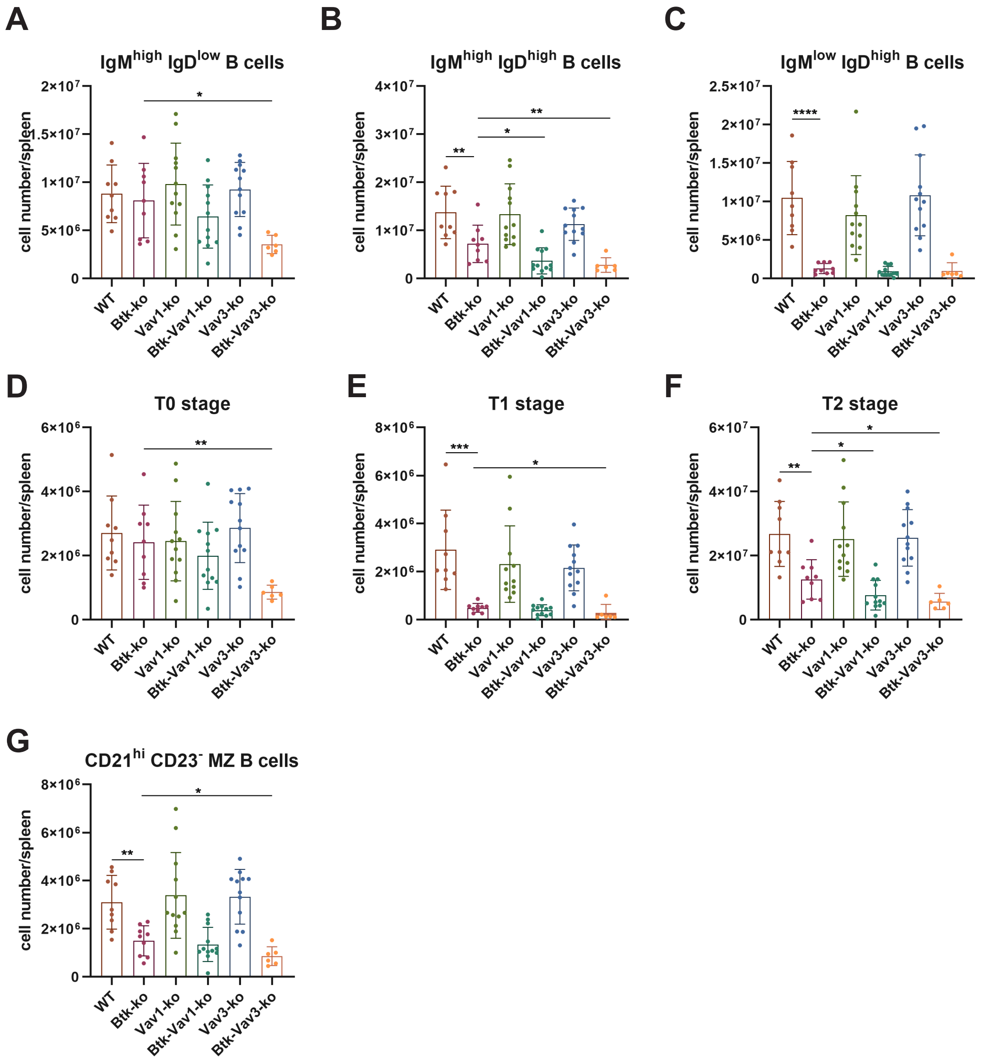

Figure S1

Statistics of flow cytometry analyses showing percentages of immature (IgMhigh IgDlow) (A), immature (IgMhigh IgDhigh) (B), mature (IgMlow IgDhigh) (C), T0 stage (IgMhigh IgD- CD23-) (D), T1 stage (IgMhigh IgD+ CD23- (E), T2 stage (IgMhigh IgD+ CD23+ (F) and MZ B (CD21high CD23-) B cells (G). Each point represents data from a single mouse. Data are merged from at least three independent experiments. *$p < 0.05$, **$p < 0.01$, ***$p < 0.001$ and ****$p < 0.0001$. P-values were determined using a two-tailed Student's t test or Mann-Whitney- U test.

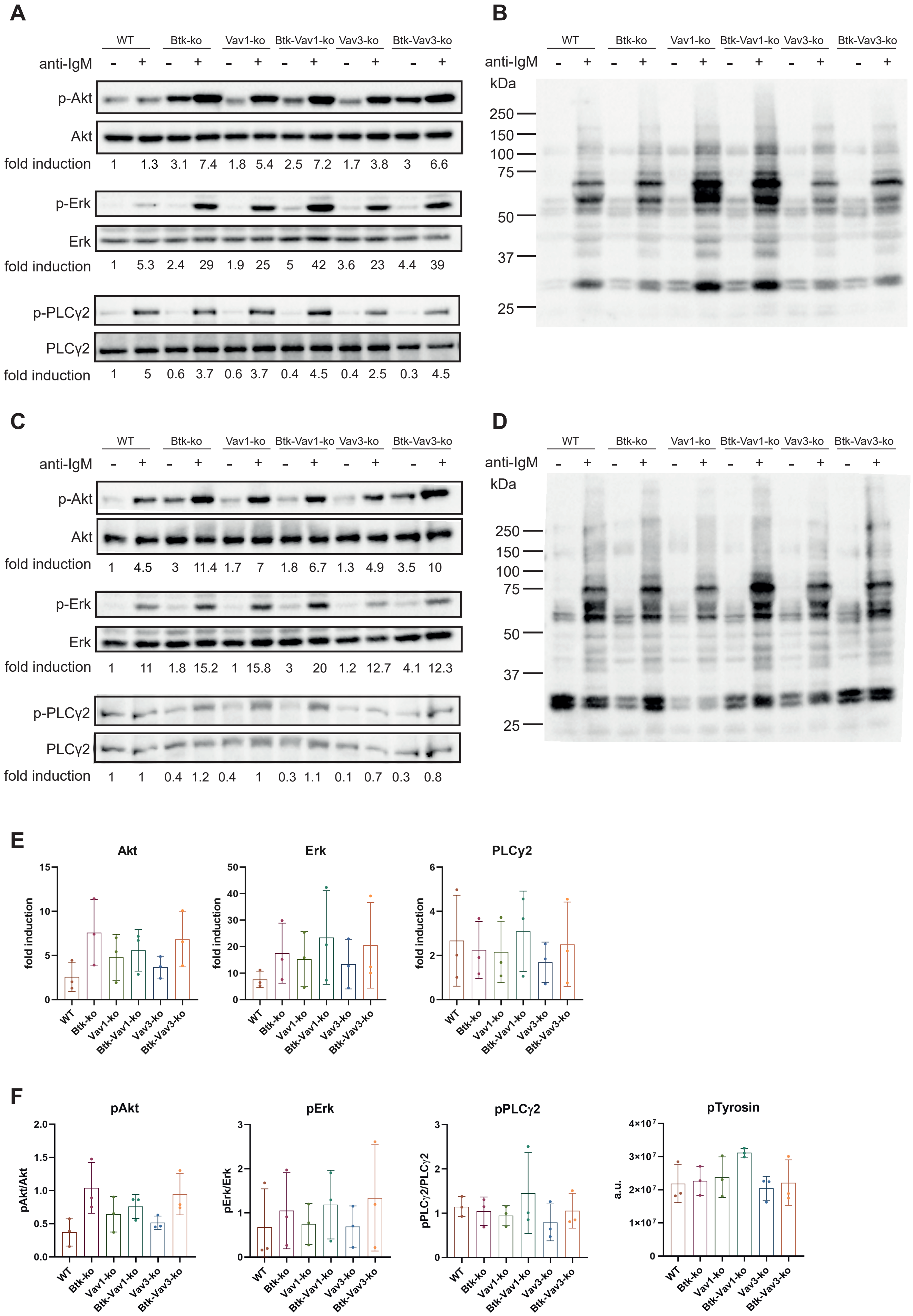

Figure S2

Naïve B cells were isolated from respective mouse strains and stimulated with anti-µ F(ab)2 for 3 min. Cells were lysed and phosphorylated forms of Akt (S473), Erk (Y204) and PLCγ2 (Y1217) as well as phospho-tyrosine were assessed by western blot. Non-phosphorylated proteins were used as loading controls (A-D). Analysis of fold induction of phosphorylated Akt, Erk and PLCγ2 in stimulated B cells compared to unstimulated B cells. (E). Quantification of pAkt, pErk, pPLCγ2 and phospho-tyrosine expression (F). Mice were analyzed in an age range from 7-14 weeks.

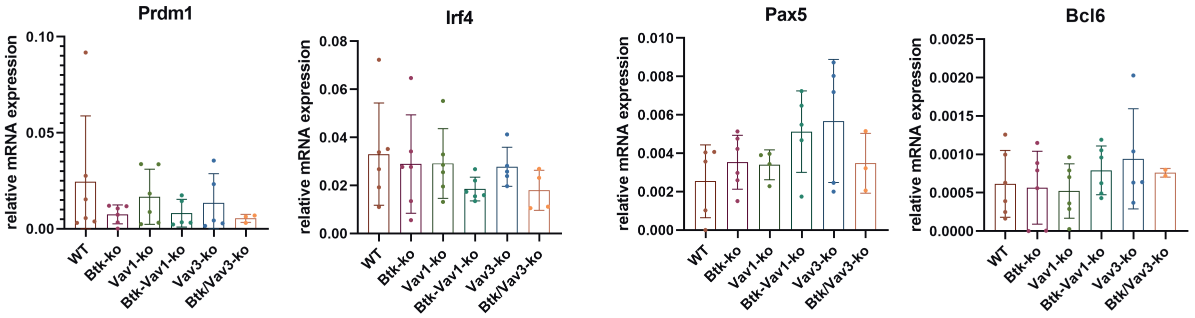

Figure S3

Naive B cells were isolated from respective mouse strains and cultured in the presence of CD40 and IL4 for 7 days. On day 7 RNA was isolated from cells and plasma cell differentiation associated transcription factors were measurd by qRT-PCR. Relative mRNA expression of Prdm1, Irf4, Pax5 and Bcl6 is shown.

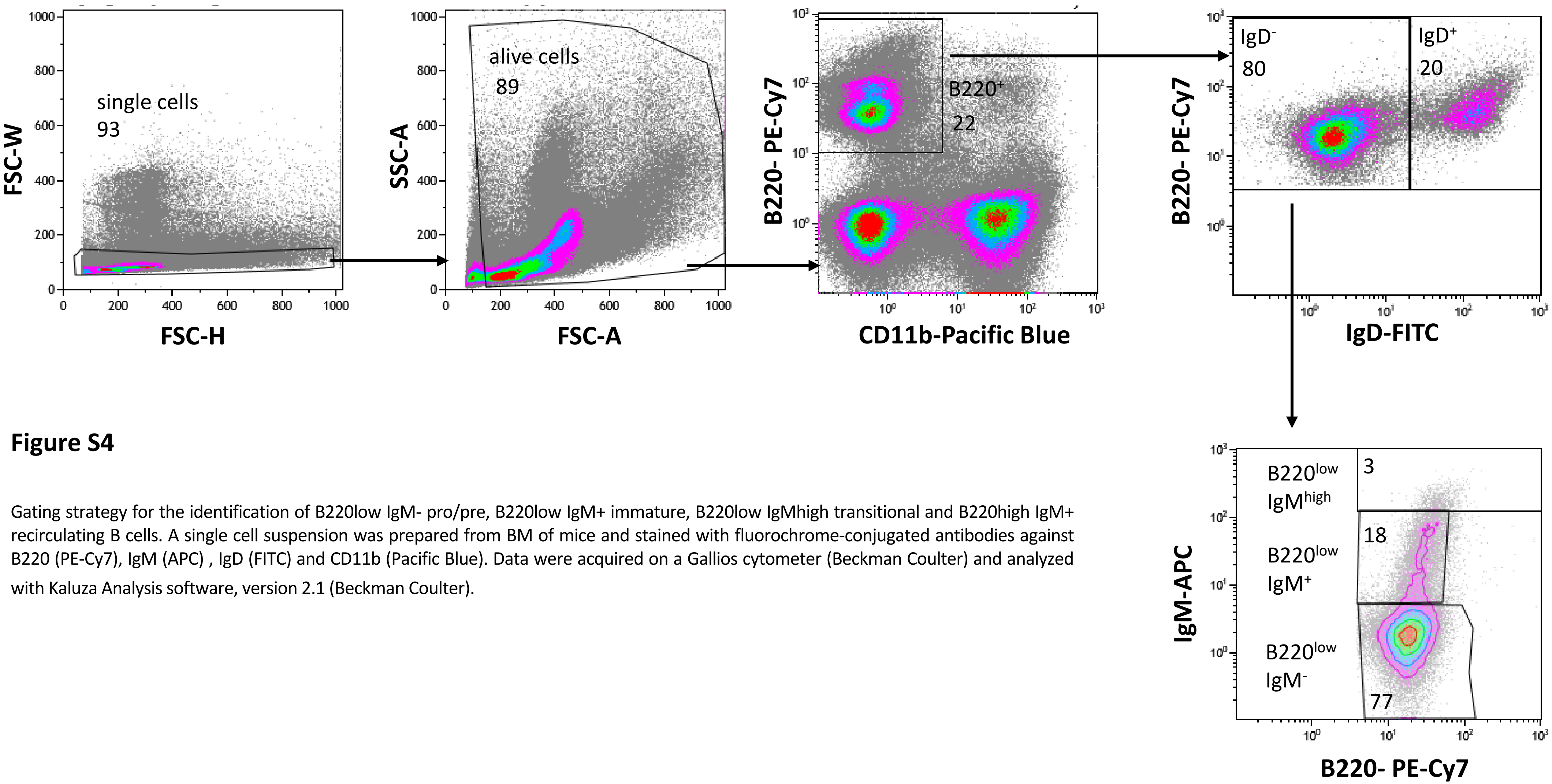

Figure S4

Gating strategy for the identification of B220low IgM- pro/pre, B220low IgM+ immature, B220low IgMhigh transitional and B220high IgM+ recirculating B cells. A single cell suspension was prepared from BM of mice and stained with fluorochrome-conjugated antibodies against B220 (PE-Cy7), IgM (APC) , IgD (FITC) and CD11b (Pacific Blue). Data were acquired on a Gallios cytometer (Beckman Coulter) and analyzed with Kaluza Analysis software, version 2.1 (Beckman Coulter).

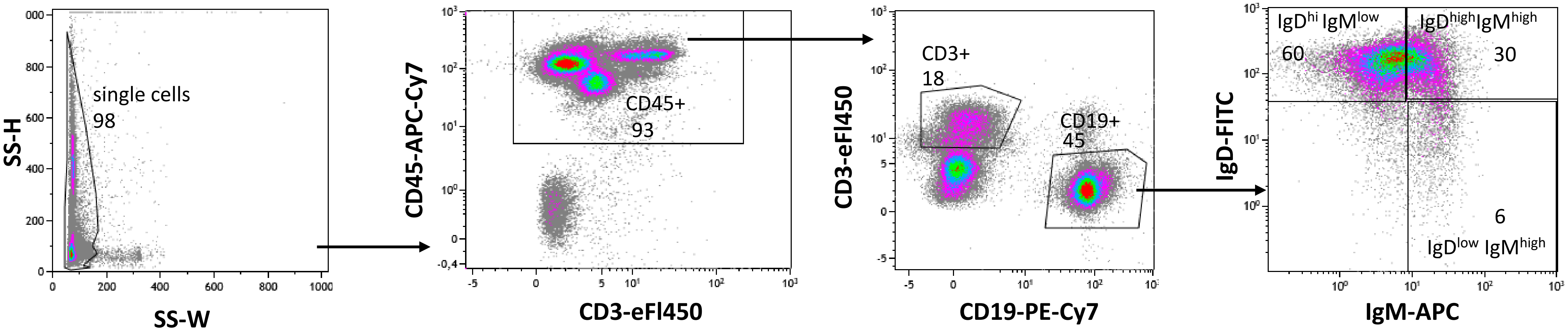

FIgure S5

Gating strategy for the identification of IgM+/IgD+ B cells. Blood of mice was stained with fluorochrome-conjugated antibodies against CD45 (APC-Cy7), CD19 (PE-Cy7), IgM (APC) and IgD (FITC). Data were acquired on a Gallios cytometer (Beckman Coulter) and analyzed with Kaluza Analysis software, version 2.1 (Beckman Coulter).

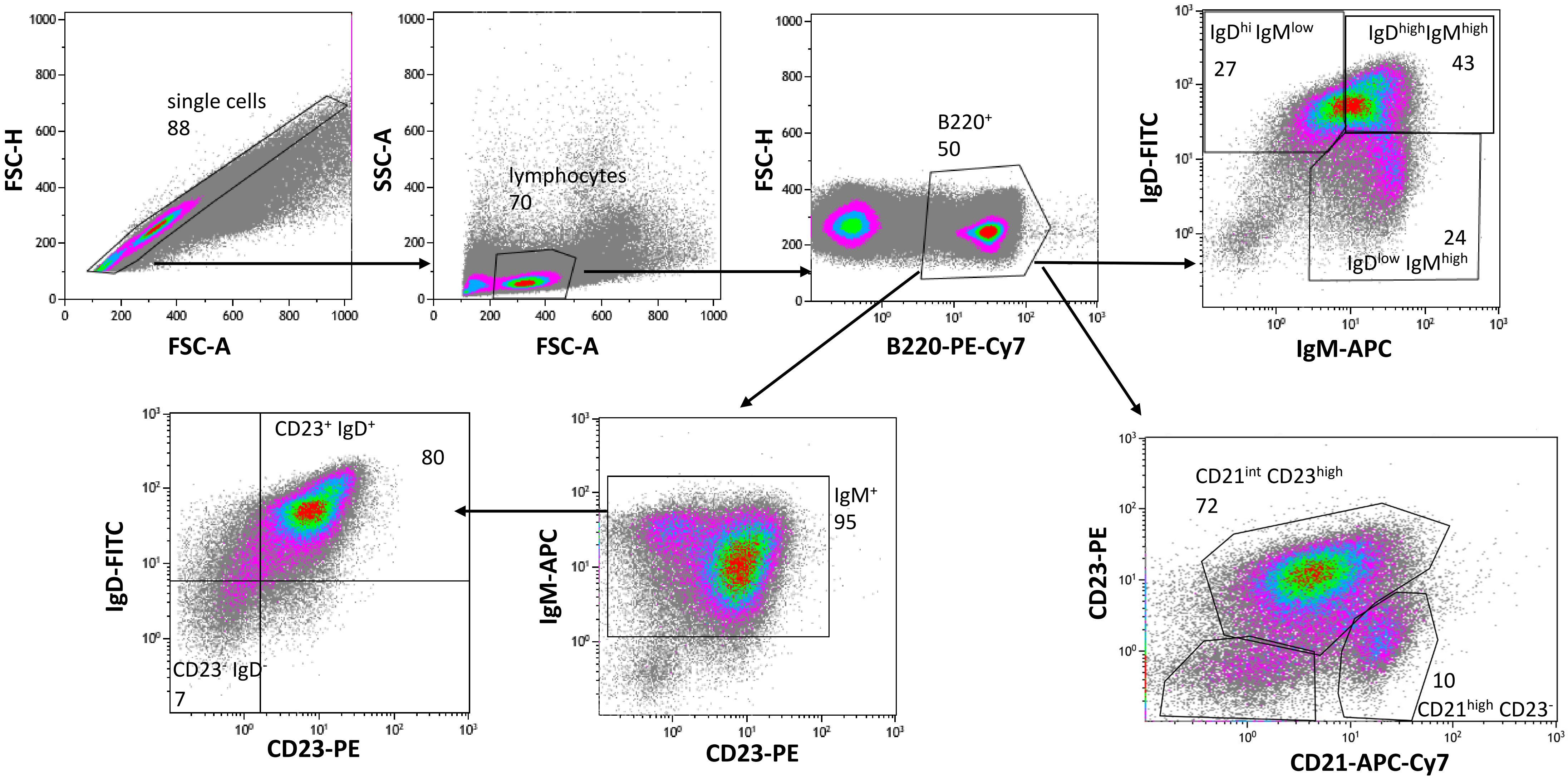

Figure S6

Gating strategy for the identification of IgM+/IgD+, MZ and T1 and T2 stage B cells. A single cell suspension was prepared from spleens of mice and stained with fluorochrome-conjugated antibodies against B220 (PE-Cy7), IgM (APC), IgD (FITC), CD23 (PE) and CD21 (APC-Cy7). Data were acquired on a Gallios cytometer (Beckman Coulter) and analyzed with Kaluza Analysis software, version 2.1 (Beckman Coulter). Lymphocytes were identified by their scatter properties (FSC-A x SSC-A plot).

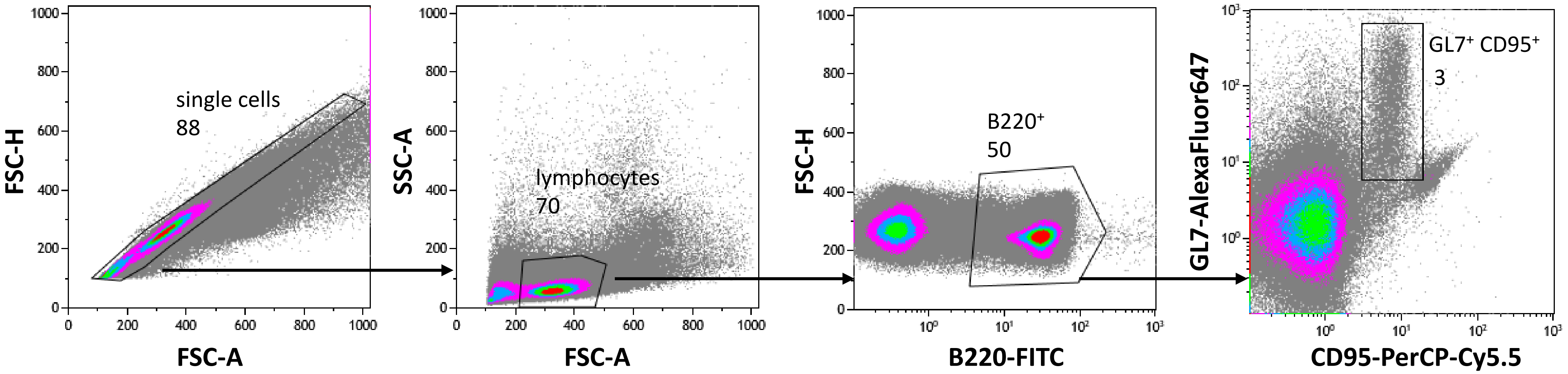

Figure S7

Gating strategy for the identification of GL7+ CD95+ GC B cells. A single cell suspension was prepared from spleens of mice and stained with fluorochrome-conjugated antibodies against B220 (FITC), GL7 (AF647) and CD95(PerCP-Cy5.5). Data were acquired on a Gallios cytometer (Beckman Coulter) and analyzed with Kaluza Analysis software, version 2.1 (Beckman Coulter). Lymphocytes were identified by their scatter properties (FSC-A x SSC-A plot).

Bone Marrow

Cell fraction	Surface markers	WT		Btk-ko		Vav1-ko		Btk/Vav1-ko		Vav3-ko		Btk/Vav3-ko	
		%	Number	%	number	%	number	%	number	%	number	%	number
**Total B cells**	$B220^+$	12 ± 3	3 ± 1	10 ± 3	2.4 ± 1.2	11 ± 3	3.4 ± 1	7 ± 2	2.2 ± 1	12 ± 3	2.5 ± 1.4	8 ± 3	2.1 ± 1.5
**Pro/pre B cells**	$B220^{low}$ IgM^- IgD^-	78 ± 2	1.7 ± 0.6	78 ± 2	1.6 ± 0.8	78 ± 3	1.7 ± 0.5	82 ± 7	1.5 ± 0.7	79 ± 2	1.4 ± 0.8	79 ± 4	1.4 ± 1
**Immature B cells**	$B220^{low}$ IgM^+ IgD^-	16 ± 2	0.5 ± 0.2	14 ± 1	0.3 ± 0.2	15 ± 2	0.5 ± 0.3	13 ± 5	0.2 ± 0.1	16 ± 3	0.2 ± 0.2	13 ± 1	0.3 ± 0.2
**Transitional B cells**	$B220^{low}$ IgM^{high} IgD^-	3 ± 1	0.2 ± 0.1	6 ± 1	0.3 ± 0.2	5 ± 1	0.2 ± 0.1	3 ± 2	0.1 ± 0.1	3 ± 0.5	0.1 ± 0.1	8 ± 2	0.2 ± 0.2
**Recirculatin B cells**	$B220^+$ IgM^- IgD^+	19 ± 3	0.6± 0.3	10 ± 2	0.2 ± 0.1	22 ± 7	0.7 ± 0.4	3 ± 2	0.03 ± 0.03	22 ± 3	0.6 ± 0.4	11 ± 4	0.1 ± 0.1

Table S1: Relative and absolute numbers of B cell subsets in the BM.

The indicated B cell subsets were gated as illustrated in Figure 1.

The mean percentage ± SD of gated cells for each genotype is shown.

The mean absolute number ± SD ($x10^6$) of cells in each subset was calculated by multiplying the percent cells in each gate by the number of splenocytes harvested from each mouse.

Blood

Cell fraction	Surface markers	WT		Btk-ko		Vav1-ko		Btk/Vav1-ko		Vav3-ko		Btk/Vav3-ko	
		%	Number	%	number	%	number	%	number	%	number	%	number
**Total B cells**	$CD19^{+}$	51 ± 7	3.6 ± 1.3	19 ± 16	1.8 ± 1.4	45 ± 12	3.0 ± 1.8	1 ± 1	0.2 ± 0.1	46 ± 8	4.1 ± 1	21 ± 9	0.8 ± 0.5
**Immature B cells**	IgM^{high} IgD^{low}	7 ± 4	0.07 ± 0.03	32 ± 5	0.4 ± 03	16 ± 4	0.08 ± 0.05	64 ± 13	0.08 ± 0.06	5 ± 1	0.04 ± 0.02	30 ± 1	0.2 ± 0.2
**Immature B cells**	IgM^{high} IgD^{high}	38 ± 9	0.2 ± 0.1	60 ± 9	1.4 ± 1.2	43 ± 13	0.2 ± 0.08	23 ± 10	0.1± 0.1	25 ± 5	0.2± 0.1	54 ± 3	0.6 ± 0.3
**Mature B cells**	$IgM^{low}IgD^{high}$	46 ± 12	3.4 ± 1.2	3 ± 2	0.1 ± 0.1	27 ± 16	3.3 ± 1.2	0.5 ± 0.7	0.002 ± 0.003	64 ± 5	3.9 ± 0.9	10± 3	0.1 ± 0.1

Table S2: Relative and absolute numbers of B cell subsets in the blood.

The indicated B cell subsets were gated as illustrated in Figure 2.

The mean percentage ± SD of gated cells for each genotype is shown.

The mean absolute number ± SD ($x10^3$) of cells in each subset was calculated by multiplying the percent cells in each gate by the number of splenocytes harvested from each mouse.

Spleen

Cell fraction	Surface markers	WT		Btk-ko		Vav1-ko		Btk/Vav1-ko		Vav3-ko		Btk/Vav3-ko	
		%	Number	%	number	%	number	%	number	%	number	%	number
**Total B cells**	$B220^{+}$	46 ± 5	38 ± 13	33 ± 6	9 ± 5	46 ± 12	26 ± 13	27 ± 9	8 ± 5	43 ± 8	32 ± 13	28 ± 7	5 ± 3
**Immature B cells**	IgM^{high} IgD^{low}	26 ± 3	8 ± 3	45 ± 4	8 ± 4	31 ± 7	9 ± 4	59 ± 9	7 ± 3	27 ± 3	9 ± 3	44 ± 10	4 ± 0.9
**Immature B cells**	IgM^{high} IgD^{high}	39 ± 2	14 ± 5	40 ± 5	7 ± 4	40 ± 4	13 ± 6	28 ± 9	4 ± 3	34 ± 4	11 ± 3	33 ± 6	3 ± 1
**Mature B cells**	$IgM^{low}IgD^{high}$	29 ± 4	10 ± 5	7 ± 2	1 ± 0.6	24 ± 8	8 ± 5	7 ± 3	1 ± 0.6	30 ± 5	10 ± 5	10 ± 6	1 ± 1
**T0 B cells**	IgM^{high} IgD^{-} $CD23^{-}$	8 ± 1	2.7± 1	14 ± 1	2.4 ± 1	8 ± 2	2.4 ± 1	18 ± 3	2.2 ± 1	8 ± 1	2.7 ± 1	12 ± 3	0.9 ± 0.2
**T1 B cells**	IgM^{high} IgD^{+} $CD23^{-}$	8 ± 1	2.9 ± 1.5	3 ± 0.5	0.5 ± 0.1	7 ± 2	2.3 ± 1.5	3 ± 1	0.3 ± 0.2	6 ± 1	2.1 ± 1	3 ± 2	0.3 ± 0.3
**T2 B cells**	IgM^{high} IgD^{+} $CD23^{+}$	80 ± 2	26± 1	74 ± 1	13 ± 6	80 ± 5	24 ± 1	67 ± 6	8 ± 4	81 ± 2	25± 8	73 ± 5	6 ± 2
**MZ B cells**	$CD21^{high}$ $CD23^{-}$	9 ± 1	3 ± 1	9 ± 1	1.5 ± 0.6	10 ± 2	3.4 ± 1.7	10 ± 2	1.5 ± 0.7	10 ± 2	3 ± 1	10 ± 1	0.9 ± 0.4

Table S3: Relative and absolute numbers of B cell subsets in the spleen.

The indicated B cell subsets were gated as illustrated in Figure 3.

The mean percentage ± SD of gated cells for each genotype is shown.

The mean absolute number ± SD ($x10^{6}$) of cells in each subset was calculated by multiplying the percent cells in each gate by the number of splenocytes harvested from each mouse.
